# Factors for Perceived Helpfulness of Support Sources Among Survivors of Intimate Partner Violence

**DOI:** 10.3390/bs15101350

**Published:** 2025-10-02

**Authors:** Hyunkag Cho, Woojong Kim, Kaytee Gillis, Kasey Goetz

**Affiliations:** 1 School of Social Work, Michigan State University, East Lansing, MI 48824, USA; gillisk6@msu.edu (K.G.); goetzkas@msu.edu (K.G.); 2 Social Work Department, University of Michigan-Flint, Flint, MI 48502, USA; kwoojong@umich.edu

**Keywords:** intimate partner violence, survivor, help-seeking, help-seeking outcomes, perceived helpfulness

## Abstract

Intimate partner violence (IPV) has far-reaching health and social consequences, particularly for survivors experiencing polyvictimization—multiple forms of IPV such as physical, emotional, and sexual abuse. This study examined help-seeking behaviors and the perceived helpfulness of formal support sources (police, medical professionals, and psychologists) among a nationally representative sample of 2387 IPV survivors drawn from the 2010 National Intimate Partner and Sexual Violence Survey (NISVS) in the United States. Latent class analysis identified three distinct polyvictimization profiles: Coercive Control and Psychological Aggression (CCPA), Psychological and Physical Violence (PPV), and Multiple Violence (MV). Survivors’ patterns of formal help-seeking varied significantly by gender, sexual orientation, socioeconomic status, and type of victimization. Psychologists were the most commonly contacted and perceived as the most helpful overall, though disparities emerged. Female survivors and those with less severe victimization were more likely to rate support as helpful, whereas male and sexual/gender minority (SGM) survivors, particularly those facing severe or multiple forms of violence, were less likely to find formal sources helpful—especially law enforcement. These findings highlight the need for more inclusive, culturally competent, and trauma-informed services tailored to the diverse experiences of IPV survivors.

## 1. Health Consequences of IPV

Intimate partner violence (IPV) is a serious public health problem, not only because it affects victims’ short-term health and well-being, but also due to the fact that it may result in longer-lasting or even life-long trauma. Many survivors suffer from a number of health concerns as well as struggles with finances and un/underemployment. Seeking help from various sources (e.g., police, doctor, family members, online support groups) is critically important to reduce such negative consequences. The literature, however, suggests that help-seeking does not always result in positive outcomes. It is not clear yet how help-seeking outcomes are associated with types of IPV victimization, types of help sources, and survivors’ gender and sexual orientation. Furthermore, previous studies often overlooked potentially cumulative effects of polyvictimization. This study addresses this gap, using a nationally representative dataset.

Experiencing IPV increases the risk of both short and long-term health consequences. Many of the short-term health consequences survivors are at risk for include physical injuries such as bruises, fractures, and concussions, as well as psychological effects like acute stress, anxiety, and depression, as well as academic and employment struggles (Clemente-Teixeira et al., 2022; Klencakova et al., 2021; Spencer et al., 2023; Wood et al., 2020). The long-term consequences that survivors of IPV may face include chronic physical conditions such as ongoing pain, gastrointestinal problems, and cardiovascular issues; persistent mental health struggles like prolonged depression, anxiety, and increased risk of self-harm (Coker et al., 2002; Janssen et al., 2021; Spencer et al., 2023; Wood et al., 2020), as well as post-traumatic stress disorder (PTSD) and substance abuse (Mehr et al., 2023; Fernández-Fillol et al., 2021). The risk of many different health consequences of IPV victimization seems to be especially high among those who have experienced polyvictimization (Banyard et al., 2020; Brewer et al., 2018). Polyvictimization is defined in this study as when a victim experiences more than one form of IPV over time such as physical, psychological, emotional, and/or sexual abuse.

## 2. Survivors’ Help-Seeking

There are many physical and mental health consequences of IPV that can significantly disrupt a survivor’s life and well-being. However, when survivors are willing and able to seek help following experiences of IPV, this greatly reduces their chances of long lasting health consequences (Renner & Copps Hartley, 2021; Wright & Johnson, 2012). Survivors seek help from various sources after IPV victimization, including from formal (e.g., police, doctor, domestic violence shelters) and informal (e.g., family, friends) sources. Formal support sources are sometimes differentiated by IPV specialists (e.g., shelters, IPV counselling programs) and generalists (i.e., those that work with people facing a variety of concerns, not just IPV; e.g., police, medical professionals, psychologists). While some staff within generalist services may have specialized IPV training, typically most do not, which can be associated with survivors’ help-seeking behaviors.

Survivors’ forms of help-seeking have been associated with many factors, such as the type of IPV victimization as well as survivor demographics. Victims experiencing physical violence seem to be more inclined to seek medical attention from formal sources like doctors or legal resources, likely due to visible injuries and a need for immediate physical care and documentation of abuse (Coker et al., 2002; Duterte et al., 2008; McCart et al., 2010; Puente-Martínez et al., 2023). Victims of emotional and psychological abuse tend to seek help from formal sources like mental health workers primarily, likely to address the emotional and psychological impact of this form of abuse (Puente-Martínez et al., 2023; Radell et al., 2021). Research indicates that victims of physical violence, including those experiencing multiple forms of violence (polyvictimization) such as physical or sexual abuse, tend to seek help more often than those who face non-physical forms of IPV, like emotional or psychological abuse (Puente-Martínez et al., 2023). Similarly, victims of repeated or severe IPV may seek assistance from formal sources such as law enforcement to obtain legal protection or intervention, particularly in cases involving physical violence or threats of harm to themselves or their children (Puente-Martínez et al., 2023). Victims who experience non-physical forms of abuse such as stalking are less likely to seek help from formal supports such as police than victims of physical or sexual abuse (Puente-Martínez et al., 2023; Wengloski & Cleary, 2024).

Research indicates significant differences in help-seeking behaviors among different genders of survivors of IPV. Male victims of IPV are frequently found to be less likely to seek help, either from formal or informal sources, than female victims (Douglas & Hines, 2011; Lysova & Dim, 2020; Lysova et al., 2022; Taylor et al., 2022). Men may feel ashamed or fear the stigma associated with being a victim, leading to delayed help-seeking or complete avoidance of services, which can lead to underreporting and lower rates of seeking help (Douglas & Hines, 2011). Similarly, men may face societal stigma and pressure to adhere to traditional masculine norms, which can inhibit their willingness to seek help from formal sources like doctors, psychologists, or the police (Douglas & Hines, 2011; Taylor et al., 2022; Tsui, 2014). These stigmas and pressures likely contribute to a lack of reporting, and thus a lack of research and data on male survivors of IPV. Conversely, research indicates that women are more likely than men to seek support from formal sources such as seeking medical attention, psychological counseling, or legal assistance following experiences of IPV, a trend that is likely attributed to greater societal acceptance and support for women in seeking help (Douglas & Hines, 2011; Tsui, 2014). However, although female survivors have a greater likelihood of seeking help, they may also encounter barriers such as victim-blaming attitudes, lack of understanding from service providers, and fear of not being believed (Gracia, 2014). Thus, across genders, victims are more likely to seek support from at least one informal source such as friends and family, where they are less likely to experience these barriers (Sylaska & Edwards, 2014).

The experiences of Lesbian, Gay, Bisexual, Transgender, and Queer or Questioning (LGBTQ+) individuals in IPV situations are often compounded by the stigma and discrimination they may face within both their intimate relationships and society at large (Bermea et al., 2021; Calton et al., 2016). Research suggests that not only is sexuality and gender identity a risk factor for IPV, but LGBTQ+ survivors may be less likely to seek help due to fears of homophobia or transphobia from service providers, which can deter them from accessing the necessary resources (Bass & Nagy, 2023; Bermea et al., 2021; Calton et al., 2016; Scheer & Baams, 2021; Wengloski & Cleary, 2024). While more research is needed on this population, stigma and discrimination seem to remain a major barrier for LGBTQ survivors during both formal and informal help-seeking (Bass & Nagy, 2023; Bermea et al., 2021; Calton et al., 2016). Although research is limited as to whether LGBTQ survivors of color differ from their White counterparts in terms of help-seeking behavior or perceptions of helpfulness, some studies do point to suggestions that Black LGBTQ survivors of abuse are more likely to seek informal support (friends, family, significant others) largely due to barriers to formal supports (like biases and discrimination and racism; Buckley et al., 2025; Butler et al., 2025).

While gender plays a significant role in shaping IPV survivors’ help-seeking behaviors, immigration status brings additional barriers for survivors of all genders. Studies on immigrant IPV survivors’ help-seeking behaviors show that these individuals are often less likely to seek formal medical or legal assistance compared to native-born survivors due to factors such as fear of deportation, language barriers, lack of trust in authorities or medical providers, and limited awareness of available services (Femi-Ajao et al., 2018; Hulley et al., 2023; Park et al., 2021; Shuman et al., 2022; Stewart et al., 2012; Thurston et al., 2013). Research shows that help-seeking behaviors of immigrant survivors remain compounded by racism, discrimination, lack of awareness or knowledge of resources, as well as socioeconomic disadvantages, all factors that likely influence their experiences of IPV and help-seeking (Hulley et al., 2023; Rodríguez et al., 2009; Satyen et al., 2019; Stewart et al., 2012).

## 3. Perceived Helpfulness of Support Sources

The available literature suggests differences in perceived helpfulness of support sources by gender. Both male and female victims of IPV face significant barriers to seeking help, but gendered expectations and resource availability shape their willingness and ability to reach out for support (Cho et al., 2020). The information available has found that female survivors are more likely to use informal sources first, often due to concerns about privacy, fear of retaliation, or distrust of formal institutions, but that formal sources of support, such as counseling, are found to be helpful in teaching coping skills and improving decision making (Ansara & Hindin, 2010b; Robinson et al., 2021). Domestic violence shelters have been a source of support for women and children fleeing abusive situations since the 1970s, and remain one of the most widely accessed and available support for survivors; however, there seems to be insufficient research on their effectiveness (Goodhand, 2017; Klein et al., 2021). One study of female survivors found that some formal sources of support, however, such as domestic violence hotlines or programs were associated with an increase in mental health symptoms such as anxiety and hopelessness, maybe due to survivors with high levels of psychological distress being more likely to reach out for formal supports (Zhang et al., 2024). The inconsistency in these findings may be related to whether service providers have specialized IPV training. Service providers without specialized IPV training may be less likely to meet specific needs of IPV survivors, resulting in less desirable outcomes.

Male IPV survivors, on the other hand, were shown to have mixed perceptions of the helpfulness of support sources. The results of some studies highlight that men are more likely to feel supported by informal sources of support, such as friends or family, rather than formal services (Huntley et al., 2019; Kim et al., 2024; Morgan & Wells, 2016). Some studies report that, for male survivors, formal sources such as law enforcement, legal services, and services specifically targeted toward domestic violence survivors, such as hotlines, were perceived as unhelpful, likely because these services are traditionally directed toward female IPV survivors (Douglas & Hines, 2011; Morgan & Wells, 2016). These findings may be related to that most domestic violence shelters are for women (transwomen included), whereas IPV counselling and outreach programs serve survivors of all genders.

Available literature shows that there are variations in perceived helpfulness of support sources by survivors’ sexual orientation. Of the survivors who contacted the police as a form of help-seeking following IPV, non-heterosexual survivors were less likely to find them to be helpful (Kurdyla et al., 2021). Similarly, LGBTQ survivors of IPV are less likely to find some formal help-seeking sources such as medical professionals and healthcare workers helpful, likely due to barriers such as stigma and discrimination that contribute to having less desirable outcomes for LGBTQ survivors (Kurdyla et al., 2021). Studies have found that LGBTQ survivors tend to prefer informal help sources and report them as more helpful such as friends and supportive people in their community over formal sources such as police or medical staff (Bermea et al., 2021; Kurdyla et al., 2021). This is likely due to barriers to seeking help, including discrimination and biases in healthcare and legal systems, which can lead to reluctance or fear in accessing support (Calton et al., 2016; Kurdyla et al., 2021; Rollè et al., 2018).

Perceived helpfulness of support sources for immigrant survivors of IPV are often shaped by a combination of cultural, social, and legal barriers that can significantly affect their ability and willingness to seek formal support. Immigrant survivors have been found to be less likely to use formal supports, likely due to heightened fear of deportation, experiences of language barriers, limited knowledge of available resources, as well as the cultural stigma surrounding IPV which can all deter individuals from seeking help (Hulley et al., 2023; Park et al., 2021; Stewart et al., 2012; Thurston et al., 2013). Studies have shown that immigrant survivors are more likely to report that informal sources of support, such as family, friends, or religious leaders, are more comfortable for them to seek support from than formal services like healthcare providers, shelters, or law enforcement (Hulley et al., 2023; Park et al., 2021; Thurston et al., 2013). However, when immigrant survivors do access formal help from culturally competent services such as those where providers speak their language, they are more likely to see these support services as helpful (Al Shamsi et al., 2020).

## 4. Current Study

Although prior research has addressed perceived helpfulness of support according to gender and sexuality, there are gaps in the literature in understanding their patterns, especially across genders, including male victims and sexual minorities, and in the context of polyvictimization. Research on male survivors of IPV is relatively sparse, and existing studies often focus on physical violence rather than other forms of IPV or even polyvictimization experiences. These gaps highlight the need to identify distinct patterns in help-seeking among IPV survivors, based on their experiences and characteristics, which is critical for developing tailored services. This study fills these gaps using national data that include male, female, and sexual and gender minority survivors and measure various forms of IPV victimization. Specially, we aim to address the following research questions: (1) What is the pattern of IPV polyvictimization among IPV survivors who sought formal help after victimization? (2) How is survivors’ formal help-seeking associated with their characteristics and IPV polyvictimization experiences? (3) How is the perceived helpfulness of support sources associated with survivors’ sexual and gender identity and IPV polyvictimization experiences?

## 5. Methods

We utilized the National Intimate Partner and Sexual Violence Survey (NISVS), which gathered data in the United States from the nationally representative sample aged 18 or older in 2010 (Black et al., 2011). The Centers for Disease Control and Prevention developed the NISVS through a federally sponsored workshop aimed at establishing data systems to track and address sexual violence, stalking, and intimate partner violence (Black et al., 2011). The NISVS study design adhered to the World Health Organization’s ethical standards for conducting interviews on experiences of violence (World Health Organization, 2001). In NISVS, 18,049 individuals were contacted by a dual-frame, stratified random-digit dialing (RDD) approach, using both landline and cell phone samples to account for the rising number of cell phone-only households (Black et al., 2011). To produce national and state-level estimates, samples were stratified by state, with additional sampling from smaller states. Weights were calculated to account for factors like different sampling rates across states and selection probabilities in both landline and cell phone frames (Black et al., 2011). For this study, we first selected survivors of IPV who experienced violence from their intimate partners including spouse, live-in partner, fiancé, boy or girlfriend, dating partner, someone seeing, and sex partner. Among 8587 IPV survivors, we selected 2387 survivors who sought help from police (*n* = 1050), medical doctors (*n* = 718), and/or psychologists (*n* = 1781).

## 6. Measurements

### 6.1. IPV Victimization

The NISVS captured lifetime victimization experiences using 60 behaviors from seven different types of violence: (1) psychological aggression (5 items such as “told you that you were a loser, a failure, or not good enough”; “told you no one else would want you”); (2) coercive control (14 items such as “tried to keep you from family or friends”; “threatened to take your children away”); (3) less severe physical violence (3 items such as “threatening to physically harm you; pushing or shoving”); (4) severe physical violence (9 items including “kicking”; “slamming”; “beating”); (5) stalking (7 items such as “making unwanted phone calls to you”; “watched you from a distance”); (6) rape (12 items; e.g., “having sex by using physical force”); and (7) non-rape sexual assaults (10 items such as exposing their body parts to you; fondling or grabbing your sexual body parts). Using 60 dichotomous variables across seven categories, we created seven continuous variables by summing the number of behaviors reported by the survivors within each category. These continuous variables were utilized to classify IPV polyvictimization, as described in the analysis section. Cronbach’s alphas for the seven scales ranged from 0.70 to 0.82 (psychological aggression: 0.74; coercive control: 0.80; less severe physical violence: 0.77; severe physical violence: 0.82; stalking: 0.70; rape: 0.79; non-rape sexual assaults: 0.79).

### 6.2. Safety Concern

The NISVS ask whether survivors were ever concerned for their safety during their IPV experiences. We created a dichotomous variable based on responses to this question by coding participants who answered ‘yes’ (indicating concern for their safety) as having safety concerns as a consequence of IPV victimization.

### 6.3. Help-Seeking and Perceived Helpfulness of Support Sources

The NISVS asked survivors if they ever talked to the following professionals: police, doctor or nurse, psychologist or counselor, and hotlines. We created three separate dichotomous variables to identify whether formal help was sought from police (Police), doctor or nurse (Medical Professional), or psychologist or counselor (Psychologist). Hotlines were excluded from the analysis as the number of respondents who contacted hotlines was too small for reliable analysis. Additionally, the NISVS used a question to capture how helpful it was to the respondents when they spoke to the professional (Police, Medical Professional, or Psychologist) regarding their victimization. The response options were: very helpful, somewhat helpful, a little bit helpful, and not at all helpful. By combining the first three categories (very helpful, somewhat helpful, a little bit helpful) into one category (helpful) and using “not at all helpful” as another (not helpful), we created three dichotomous variables to represent the perceived helpfulness of each professional.

### 6.4. Survivors’ Sex and Sexual Orientation

The NISVS included two variables about sexual and gender identity and orientation. One variable indicated biological sex (Male and Female). The other question asked about sexual and gender identity and orientation by four categories: heterosexual or straight, gay or lesbian, bisexual, and transgender. We reduced it to two categories: heterosexual/straight (Heterosexual) and sexual and gender minority (SGM).

### 6.5. Demographic Characteristics

We included five demographic and socioeconomic characteristics of the survivor as control variables. Respondents’ race was captured with seven categories: White, Black, Asian, Native Hawaiian and Pacific Islander, American Indian and Alaska Native, Other Race, and Multiracial. Due to small sample sizes in non-White and non-Black subgroups, these were consolidated into three categories: White, Black, and Other Races. In addition, based on the information from another question about Hispanic origin, respondents were reclassified into four groups: non-Hispanic White, Black, Hispanic, and Other Races. Education level, which included eight categories from no formal education to postgraduate, was simplified into a dichotomous variable with two groups: less than high school graduation and high school graduation or more. Household income, which was captured by eight categories from under $10,000 to $75,000 or more, was recoded into two groups based on the 2010 federal poverty line: below $20,000 and $20,000 or higher. Nativity was assessed by asking if respondents were born in the U.S. (including U.S. territories and military bases), resulting in two categories: U.S.-born and foreign-born. Age was assessed as a continuous variable.

## 7. Analysis

We conducted a latent class analysis in order to capture respondents’ polyvictimization experiences based on the seven IPV types. This approach allowed us to classify respondents by their experiences of multiple forms of IPV, rather than by single types of victimization. The analysis was conducted in two steps. First, IPV polyvictimization categories were created by latent class analysis (LCA) based on seven continuous variables of IPV victimization (e.g., psychological aggression, severe physical violence, rape) using Mplus v.7 (Muthén & Muthén, 2012). LCA identifies substantively meaningful unobserved groups, often referred to as profiles or classes, based on a set of continuous indicators (Vermunt & Magidson, 2002). The analysis was conducted as follows. Starting with a one-class model (Model 1), multiple models were generated by increasing the number of classes that were supposed to share similarities in their polyvictimization experiences until statistically and practically proper solutions were not obtained. More specifically, Lo–Mendell–Rubin adjusted likelihood ratio test (LMR; Lo et al., 2001) provided the *p* value which shows whether one model (k) is statistically better than its neighbor class (k−1) model (Nylund et al., 2007). A series of models were generated by adding an extra class until the *p* value of LMR test was greater than 0.05, which indicates there was no improvement in fit by adding more classes. Once model generation was done, we compared the information criteria model fit statistics across models, including Akaike Information Criteria (AIC), Bayesian Information Criterion (BIC), adjusted BIC, and entropy measure. Lower values on AIC, BIC, and adjusted BIC indicate a better fit. If the entropy was higher than 0.8, indicating 80% of the individuals were relevantly classified in latent classes, we considered it sufficiently high (Clark & Muthén, 2009). Also, we considered the model not promising if its smallest class included less than 5% of the sample (Bauer, 2022). After selecting the optimal model, we examined each class generated by the model, focusing on the characteristics of each class based on victimization experiences. LCA provides the average number of victimization experiences by type within each class. To describe the diverse IPV experiences across classes, the average number of behaviors in each category was reported.

After determining IPV polyvictimization class membership, which reflected different patterns of IPV experiences, we converted the latent variables into observed variables in SPSS. Chi-square analyses were then conducted to examine the characteristics of help-seeking survivors, including sex, sexual orientation, demographics, socioeconomic status, having safety concerns, and perceptions of helpfulness of each type of professional help. Next, we performed three separate multiple logistic regression analyses to assess the perceived helpfulness of each of the three professionals (Police, Medical Professional, and Psychologist) based on the type of polyvictimization and survivors’ sex and sexual orientation, controlling for other variables. All analyses were conducted using SPSS v.29 Complex Sample Analyses, taking into account the sample strata and weights in NISVS (Black et al., 2011).

## 8. Results

A latent class analysis identified the optimal number of latent classes of IPV polyvictimization reported by 2387 respondents. Table 1 presents the fit statistics. Based on the *p*-value of the LMR, five models were generated. The fit statistics, including AIC, BIC, and adjusted BIC, continued to decrease up to the fifth model, suggesting that Model 5 provided the best statistical fit. Entropy values were acceptable for all models, exceeding 0.9. However, when considering the size of the smallest class, Model 3 proved more appropriate. The smallest class in Model 3 accounted for 5.3% of the sample (*n* = 127), which was sufficient for subsequent analysis, while the smallest classes in Model 4 (2.4%, *n* = 58) and Model 5 (2.3%, *n* = 56) were too small for further analysis. Additionally, comparisons of Model 4 and Model 5 with Model 3 revealed that the newly created classes did not exhibit distinctly unique characteristics of victimization. For instance, a new class generated in Model 4 was nearly identical to an existing class, differing only in one type of IPV. As a result, Model 3 was determined to be the optimal solution for the analysis.

**Figure 1:**
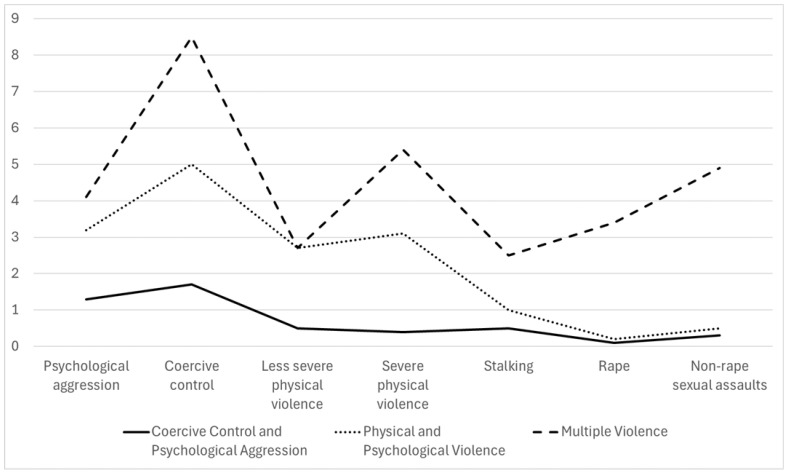
Characteristics of 3-class model.

Figure 1 presents the characteristics of each class in Model 3, showing the mean for each type of IPV. The first class was defined by experiences of psychological aggression (M = 1.3) and coercive control (M = 1.7), with minimal experiences of other IPV types, as indicated by means ranging from 0.1 to 0.5. This group, labeled as Coercive Control and Psychological Aggression (CCPA), included 58.1% of survivors (*n* = 1387). The second class, comprising 36.6% of survivors (*n* = 873), was characterized by multiple experiences of both psychological and physical violence behaviors, including psychological aggression (M = 3.2), coercive control (M = 5.0), less severe physical violence (M = 2.7), and severe physical violence (M = 3.1). They also experienced single stalking behavior (M = 1.0). This group was labeled as Psychological and Physical Violence (PPV). The third class, labeled as Multiple Violence (MV; 5.3%, *n* = 127), involved multiple experiences across all types of IPV. This group reported the highest frequencies across all types of IPV such as coercive control (M = 8.5), severe physical violence (M = 5.4), non-rape sexual assault (M = 4.9), and psychological aggression (M = 4.1). Averages for other types of violence ranged from 2.5 for stalking to 3.4 for rape.

Table 2 provides an overview of the characteristics of the 2387 survivors who sought help from the Police, Medical Professional, or Psychologist, along with the results of the chi-square analysis. Using the sample strata and weights from the NISVS, weighted percentages and unweighted sample sizes were reported. About three-fourths of the survivors were White (74.7%), followed by Hispanic (10.8%), Black (9.9%), and Other Races (4.7%). The majority of survivors were female (70.1%), while less than a third were male (29.9%). 8.3% reported their education level as less than high school education. Slightly less than a quarter (22.4%) had an annual income below $20,000. Fewer than 10% were born outside the U.S., and 8.4% were identified as SGM. More than half (51.6%) reported having safety concerns. More than three-fourths (76.1%) of survivors reported that the professionals they reached out to were helpful. On average, survivors were 45.73 years old. Out of 2387 survivors, 45.4% sought help from Police, 28.1% from Medical Professional, and more than three-quarters (76.1%) from Psychologists. Since some survivors sought assistance from multiple professionals, these groups were not mutually exclusive. White survivors contacted Psychologists (80.7%) more than Police (66.5%), while Black survivors talked to Police (16.0%) more than Psychologists (6.0%). Psychologists were the least used source of support for Hispanic survivors (8.3%). Psychologists were the most used help source among male survivors (32.3%) but the least used among female survivors. Survivors with an education level less than high school contacted Police (11.3%) more than Psychologists (6.1%). Likewise, survivors with an annual income below $20,000 contacted Police (27.9%) more than Psychologists (20.4%). Help-seeking patterns also varied based on the severity and type of victimization. Survivors who had safety concerns contacted Police (73.4%) and Medical Professionals (66.6%) more than Psychologists (45.0%). Survivors of CCPA contacted Psychologists (63.9%) more than Police (38.6%) and Medical Professionals (42.4%), while survivors of PPV and MV talked to Police (51.8% and 9.5%, respectively) and Medical Professionals (48.4% and 9.2%, respectively) more than Psychologists (31.4% and 4.8%, respectively). Survivors perceived Psychologists and Medical Professionals as more helpful (90.7% and 87.6%, respectively) than Police (63.6%).

Table 3 presents the results of three multiple logistic regression analyses, with each of the help sources as the dependent variable, highlighting the associations among the perceived helpfulness of various professionals, IPV polyvictimization, and survivors’ sex and sexual orientation. Among survivors who contacted Police, SGM survivors were less likely to perceive Police as helpful (OR = 0.31, *p* = 0.013); female survivors were more likely to find Police helpful (OR = 2.04, *p* = 0.005); and survivors of MV and PPV were less likely to perceive them as helpful compared to those of CCPA (OR = 0.61 and OR = 0.35, respectively). Among survivors who sought help from Medical Professionals, survivors who were foreign-born or had safety concerns as a result of victimization (*p* = 0.006) were more likely to perceive them as helpful (OR = 13.45 and OR = 2.74, respectively), while PPV survivors were less likely to find them helpful (OR = 0.43, *p* = 0.023). Talking to Psychologists, female survivors were more likely to perceive them as helpful (OR = 3.36, *p* < 0.001).

## 9. Discussion

The study results demonstrate that IPV survivors experience several distinctive forms of polyvictimization (Research Question [RQ] 1). The most prevalent form of polyvictimization was Coercive Control and Psychological Aggression (CCPA), followed by Psychological and Physical Violence (PPV) and Multiple Violence (MV), the most severe form of polyvictimization that involves all types of IPV. This result is consistent with the literature that shows psychological, emotional, and/or verbal IPV is the most frequently reported form (e.g., Ansara & Hindin, 2010a; Beck et al., 2013; Villamil Grest et al., 2018).

Of the three help sources examined, mental health workers such as psychologists were the most frequently used, but not without variations by the survivor’s demographic characteristics (RQ 2). White survivors were most prevalent among those who contacted a psychologist (80.7%), with Hispanic survivors being the least likely to contact them for mental health support (8.3%). This may be due to potential differences between the two groups in their attitude toward, accessibility to, and/or affordability of mental health services (Hulley et al., 2023; Park et al., 2021; Stewart et al., 2012; Thurston et al., 2013). Relatedly, psychologists were least used by Hispanic, Black, less educated, or lower income survivors. These results may also show that factors such as affordability, accessibility, and availability of psychologists may matter greatly to Hispanic, Black, less educated, and lower income survivors during the help seeking process. For these survivors, police, due to being free to access, may be one of the very few resources they can turn to for support following IPV. Furthermore, minority and/or low income survivors may prefer other ways to cope in the aftermath of their experience, including informal networks such as family, friends, those in their community, and other informal support (Hulley et al., 2023; Sylaska & Edwards, 2014). 

There were also gender differences. Psychologists were the most used help source among male survivors (32.3%) but the least used among female survivors (RQ 2). This may show that male survivors think that psychologists are more accessible than law enforcement or medical support. These results make sense, as current literature shows that law enforcement/police and medical support may not have a good understanding of males being victimized by IPV, or may have assumptions or stereotypes that assume IPV is mostly for female victims (Douglas & Hines, 2011; Gracia, 2014; Tsui, 2014). Severity of abuse also seems to matter for help-seeking behaviors; survivors who had safety concerns contacted Police and Medical Professionals more than Psychologists. Likewise, survivors of MV and PPS were found to have used Police and Medical Professionals more than Psychologists, showing that these survivors may suffer from an injury and have immediate support needs.

Overall, 76.1% of respondents found their help-seeking helpful, with mental health support such as psychologists being found to be the most helpful. However, significant gender disparities were found, as female survivors perceived psychological support as far more helpful than male survivors did (RQ 3). This gap likely stems from gendered stigma that discourages men from disclosing IPV victimization due to a combination of stereotypes that don’t often see men as able to be victims of IPV, as well as cultural expectations of masculinity often equate vulnerability with weakness (Cho et al., 2020). Additionally, psychologists may inadvertently employ interventions tailored to women’s experiences, leaving male survivors feeling misunderstood or underserved.

Furthermore, police as a help seeking source were consistently rated as the least helpful support source across all survivor groups, with some notable disparities: female survivors perceived police as marginally more helpful than male survivors, likely due to entrenched stereotypes framing IPV as the stereotype of female victim/male perpetrator in legal systems, which likely contributes to law enforcement’s lack of training to identify or support male victims (RQ 3; Cho et al., 2020; Douglas & Hines, 2011; Tsui, 2014). Not surprisingly, SGM survivors perceived police as less helpful than non-SGM survivors, likely due to police and law enforcement having a history of biased views against male and SGM survivors (Douglas & Hines, 2011; Morgan & Wells, 2016). Notably, survivors of PPV and MV found police less helpful than those experiencing CCPA. This may be due to officers often overlooking non-physical abuse that doesn’t align with conventional IPV narratives centered on gender stereotypes, as well as stereotypes about visible physical or sexual violence. These gaps imply that there may be systemic failures in society’s ability to recognize subtler abuse forms or address the unique barriers male and SGM survivors face when seeking support following IPV.

These findings should be viewed within the context of study limitations. First, due to the cross-sectional design of the NISVS, it was not possible to determine causal or temporal links between polyvictimization types and help-seeking behavior. Future research in this field would benefit from longitudinal studies that include comparison groups. Second, because the data were gathered retrospectively, there is potential for errors that could not be controlled by researchers. This study used the NISVS collected in 2010. Although the dataset includes the most comprehensive IPV variables available to date, it may be outdated, given that since 2010, social norms regarding IPV, perceptions of the severity of IPV, and public discourse may have shifted considerably. As a result, while the findings highlight patterns of IPV and their associations with help-seeking, they may not fully capture the current dynamics of IPV and should be cautiously interpreted within the historical context of the data collection period. Third, although the NISVS study design followed the World Health Organization’s ethical standards for conducting interviews on experiences of violence, the survey did not capture whether respondents were alone during the interview. Therefore, we could not rule out the possibility of reporting bias due to the presence of others during data collection. Fourth, the dataset did not include contextual characteristics such as the presence of children or social norms (e.g., justification of IPV and prevalence), which limits our ability to disentangle mechanisms underlying IPV. In addition, safety concerns were measured dichotomously, which may not capture the full complexity or varying degrees of survivors’ concerns. Another limitation is that the data include only certain types of professionals as sources of help-seeking. Therefore, the findings cannot be generalized to survivors who sought support from other professional or specialized services. Future research needs to examine a broader range of professionals and service providers, such as advocates and community-based organizations, to capture a more comprehensive picture of help-seeking and perceived helpfulness. Lastly, the dataset did not adequately represent several subpopulations. For example, individuals who were neither White nor Black were categorized as “Other,” and diverse sexual and gender minority groups were grouped together under “Sexual minority.”

## Figures and Tables

**Table 1:** Goodness of Fit Statistics (*N* = 2387).

Model Tested	Loglikelihood	AIC	BIC	Adjusted BIC	Entropy	Relative Frequency for Smallest Class	*p*-Value LMR Adjusted LRT
1-Class	−31,283	62,595	62,676	62,631	-	-	-
2-Class	−28,987	58,017	58,144	58,074	0.90	29.1%	<0.001
3-Class	−27,393	54,846	55,020	54,924	0.93	5.3%	0.007
4-Class	−26,688	53,452	53,672	53,551	0.94	2.4%	0.033
5-Class	−26,197	52,487	52,753	52,607	0.93	2.3%	0.185

AIC = Akaike Information Criterion; BIC = Bayesian Information Criterion; LMR adjusted LRT = Lo–Mendell–Rubin (LMR) adjusted likelihood ratio test.

**Table 2:** Survivor characteristics by types of help sought.

	Total (100%, 2387)	Police (45.4%, 1050)	Medical Professional (28.1%, 718)	Psychologist (76.1%, 1781)
			Weighted % (Unweighted n)	
*Race*				
White	74.7% (1884)	66.5% (758) *	74.4% (574)	80.7% (1478) *
Black	9.9% (188)	16.0% (136) *	10.2% (47)	6.0% (88) *
Hispanic origin	10.8% (154)	13.2% (75)	11.7% (51)	8.3% (104) *
Other Races	4.7% (161)	4.3% (81)	3.7% (45)	5.0% (111)
*Sex*				
Male	29.9% (654)	20.7% (219) *	23.1% (150) *	32.3% (505) *
Female	70.1% (1733)	79.3% (831) *	76.9% (568) *	67.7% (1276) *
Education: less than High School	8.3% (156)	11.3% (92) *	8.6% (49)	6.1% (89) *
Income: less than 20 k	22.4% (460)	27.9% (259) *	24.5% (162)	20.4% (305) *
Foreign-Born	9.8% (204)	10.7% (101)	11.4% (72)	8.5% (141)
SGM	8.4% (151)	6.3% (48)	8.0% (31)	9.2% (126)
Having Safety Concerns	51.6% (1229)	73.4% (748) *	66.6% (483) *	45.0% (848) *
*Polyvictimization Type*				
CCPA	58.7% (1387)	38.6% (400) *	42.4% (300) *	63.9% (1103) *
PPV	35.9% (863)	51.8% (557) *	48.4% (341) *	31.4% (577) *
MV	5.2% (127)	9.5% (93) *	9.2% (77) *	4.8% (101)
Helpful	76.1% (1854)	63.6% (686) *	87.6% (622) *	90.7% (1623) *
		Mean (SE)		
Age		45.73 (0.64)	48.10 (0.77)	46.52 (0.49)

Note: Asterisks indicate statistically significant differences among those who sought a specific type of help, with *p*-values adjusted using the Bonferroni method. CCPA = Coercive Control and Psychological Aggression, PPV = Psychological and Physical Violence, MV = Multiple Violence.

**Table 3:** Multiple Logistic Regression Analysis Results of the Associations among Perceived Helpfulness of Professionals, IPV Polyvictimization, and Survivors’ Sex and Sexual Orientation.

	Police	Medical Professional	Psychologist
	Odds Ratio (95% CI)		
Black vs. White ^a^	1.05 (0.60–1.84)	0.94 (0.28–3.19)	0.95 (0.35–2.57)
Hispanic vs. White	1.79 (0.80–3.99)	2.19 (0.53–9.07)	1.36 (0.46–3.98)
Other Races vs. White	1.46 (0.51–4.20)	0.84 (0.10–6.89)	0.37 (0.12–1.15)
Income: Less than 20 k	1.03 (0.65–1.62)	0.62 (0.30–1.31)	0.78 (0.40–1.50)
Less than high school	0.84 (0.43–1.64)	0.54 (0.18–1.58)	1.18 (0.42–3.30)
SGM	0.31 (0.12–0.78) *	1.18 (0.28–4.97)	2.01 (0.76–5.33)
Foreign-born	0.77 (0.32–1.84)	13.45 (1.23–146.72) *	1.53 (0.58–4.08)
Female	2.04 (1.24–3.38) **	1.39 (0.63–3.05)	3.36 (1.80–6.29) ***
PPV vs. CCPA ^a^	0.61 (0.40–0.92) *	0.43 (0.20–0.89) *	0.91 (0.52–1.60) ^b^
MV vs. CCPA	0.35 (0.16–0.75) *	0.47 (0.15–1.52)	-
Having Safety Concerns	1.39 (0.85–2.25)	2.74 (1.34–5.59) *	0.75 (0.39–1.42)
Age	0.99 (0.98–1.01)	1.01 (0.98–1.04)	0.99 (0.97–1.01)

Dependent variable: Perceived helpfulness of each professional help source. ^a^ Reference categories: CCPA, White. ^b^ Odds ratios were calculated for those who experienced both PPA and MV. As 99% of survivors (100 out of 101) who experienced MV reported psychologists as helpful, we combined PPA and MV and included the variable to the model. * *p* < 0.05, ** *p* < 0.01, *** *p* < 0.001.

## Data Availability

Study data is not available to others as it is restricted only to authorized researchers.
